# Multifaceted Analysis of the Thermal Properties of Shielding Cement-Based Composites with Magnetite Aggregate

**DOI:** 10.3390/ma17122936

**Published:** 2024-06-15

**Authors:** Roman Jaskulski, Krzysztof Liszka, Daria Jóźwiak-Niedźwiedzka

**Affiliations:** 1 Department of Civil Engineering, Wrocław University of Environmental and Life Sciences, 50-375 Wroclaw, Poland; 2 Institute of Fundamental Technological Research, Polish Academy of Sciences, Pawińskiego 5b, 02-106 Warsaw, Poland; djozwiak@ippt.pan.pl

**Keywords:** shielding concrete, thermal properties, magnetite aggregate

## Abstract

The paper presents and discusses the results of a study of the thermal properties of cement composites with different contents of magnetite aggregate (0%, 20%, 40% and 60% by volume). The effect of grain size on the evaluated thermal properties was also investigated. For this purpose, concrete containing 50% by volume of magnetite aggregate with four different fractions (1–2 mm, 2–4 mm, 4–8 mm and 8–16 mm) was used. Thermal parameters were evaluated on specimens fully saturated with water and dried to a constant mass at 65 °C. The series with varying grain sizes of magnetite achieved thermal conductivity values in the range of 2.76–3.03 W/(m·K) and 2.00–2.21 W/(m·K) at full water saturation and after drying to a constant mass, respectively. In the case of the series with 20% magnetite by volume, the thermal conductivity was 2.65 W/(m·K) and 1.99 W/(m·K) for the material fully saturated with water and dried to a constant mass, respectively. The series with a 60% volume share of magnetite obtained values of this parameter of 3.47 W/(m·K) and 2.66 W/(m·K), respectively, under the same assumptions.

## 1. Introduction

Determining the thermal properties of composite materials with a heterogeneous macroscopic structure, consisting of several phases with significantly different thermal parameters, is a major challenge. While volumetric heat capacity and specific heat are additive parameters, allowing their values to be calculated with satisfactory accuracy from the composite’s composition when knowing these thermal parameters for each of the constituent phases, conductivity and thermal diffusivity do not show such a simple relationship. There is no model that can accurately calculate these values. The models known from the literature, e.g., the series model, parallel model, Lichtnecker model or Maxwell model [1], only allow a rough estimation or limitation of the range within which these parameters fall.

None of the existing theoretical models consider the size of the clusters of individual phases that make up a complex composite, such as mortar or concrete. Arguably, in most cases, this is not a relevant parameter, but in the case of the material studied for this research, which is a cementitious composite with magnetite aggregate, this cannot be assumed a priori. Due to the significant difference in the thermal parameters of magnetite and cement mortar based on siliceous sand, the results obtained make it possible to observe the influence of using varying amounts of magnetite aggregate, as well as different fractions of it, on the formation of the thermal parameters of a shielded cement-based composite with magnetite aggregate.

The influence of the analysed factors and the level of their significance can only be identified through experimental research because they are shaped by factors that are difficult to define in numerical modelling, e.g., the workability of the mixture. The workability of the mix is complexly dependent on the composition of the mix while significantly affecting the distribution of the aggregate in the hardened cementitious composite and its porosity. These are parameters that have a significant impact on the coefficient of thermal conductivity [2].

This is the distinction and relevance of the research carried out, demonstrating clear differences in the thermal parameters of concrete in cases where, theoretically, they should not exceed the standard error of measurement, because, according to theoretical models, the cases studied do not differ from each other in a way that should affect the values of their thermal parameters (with the same volume proportions of materials with different values of thermal parameters). The novelty of the present work lies primarily in the comprehensive study of the effect of the grain size of the highly thermally conductive dispersed phase on the thermal performance of a composite in which the dispersed phase has a significantly lower conductivity. Magnetite was used as the dispersed phase, and the dispersed phase is a cement-sand matrix. At the same time, the study was not limited to investigating thermal conductivity alone. No results of this type of experimental study are available in the literature.

Of course, the research presented here is not suspended in a scientific void, as evidenced by publications containing the results of studies of similar materials or similar issues. The influence of magnetite aggregate size, albeit in relation to other parameters of cementitious composites, was the subject of a study by Sadrmomtazi et al. [3], who focused primarily on mechanical parameters, whose relationship with the degree of fineness of the material seems more obvious. They have found that with increasing maximum aggregate size, there is an increase in fracture energy and ductility. Mechanical parameters were also studied by Lotfi-Omran et al. [4] who, however, also considered the less obvious relationship between aggregate grain size and the shielding properties of concrete with magnetite aggregate. Their results indicated that increasing the maximum aggregate size reduced passing radiation flux and improved the shielding characteristics of the heavyweight magnetite concrete. The influence of various parameters on the thermal performance of concrete has been studied, among others, by Khan [5]. Of his several conclusions, the most interesting one seems to be that the thermal conductivity of concrete increases more with increasing moisture content in the range of moisture change from 0–50% than above this range. Studies of concrete with high thermal conductivity aggregate have been carried out by Kim et al. [6]. According to their research, the main factors affecting the conductivity of concrete are the volume content of the aggregate and the moisture condition of the specimen.

The research presented in this paper combines two areas, which include the investigation of the thermal performance of cementitious composites (as in papers [7,8]) and the investigation of heavy shielding concrete. The results of related studies combining these scientific areas can be found in papers [1,9,10,11,12], among others. However, all these studies did not include the effect of aggregate fineness in the material. Studies of this type of issue, which, however, only analysed the effect of the addition of nanomagnetite, can be found in papers published by, among others, Sikora et al. [13,14], Mansouri et al. [15] and Bolhassani et al. [16].

Related research areas have included studies using calorimetry to optimise the composition of heavy concrete [17] or to analyse the influence of heavyweight aggregate on cement hydration [18], as well as the effect of elevated temperatures on concrete with magnetite aggregate [19,20,21]. Studies on the various parameters of heavy concrete, to which the cementitious composite that is the subject of this paper belongs, also include works [22,23,24,25], which, however, did not contain a component related to the effect of aggregate fineness. Thus, this issue represents a certain gap in knowledge, which the present work aims to address.

Thermal property tests were conducted using a non-stationary method. The test plan was to study two parameters independently of each other. The first parameter was the amount of magnetite aggregate used, which at the same time accounted for all the coarse aggregate in the composite. The relative proportions of the aggregate fractions remained constant. The second parameter was the grain size of the magnetite aggregate. In the series that were used to investigate the influence of this parameter, the volume of the aggregate was taken as a constant, and a different aggregate fraction was adopted in each series. A detailed description of the test programme is provided later in the paper.

## 2. Materials and Methods

### 2.1. Materials

Cement-based composites were prepared consisting of siliceous river sand (density 2.65 g/cm^3^) of the fraction 0–2 mm and fineness modulus 1.96, crushed magnetite (density 4.80 g/cm^3^) divided into four fractions: 1–2 mm, 2–4 mm, 4–8 mm and 8–16 mm, and Portland ash cement CEM II/B-V 32.5 N (Cement Ożarów S.A., Ożarów, Poland). It was decided to use a cement with a lower carbon footprint than the normally used Portland cement CEM I. Tap water was used as the mixing water. One mix required the use of a superplasticiser. A w/c ratio of 0.5 was assumed for all the mixes. The proportions of the constituents in each mixture are given in Table 1.

Two groups of mixed series were prepared. One group of the series used the same proportions of ingredients, and the individual mixes differed only in the fraction of magnetite aggregate used. This aggregate accounted for 50% of the volume of the mixtures prepared (and 66.2% by weight). The mixtures in this group were designated by the letters CM and the identification of the magnetite aggregate fraction used.

The second group of series comprised mixtures in which magnetite aggregate of all four fractions was used (except for the series designated CM0, where no magnetite aggregate was used at all). The magnetite aggregate accounted for 20%, 40% or 60% of the mix volume, and the proportions of the other components remained constant. These series were also denoted by the letters CM and a number indicating the percentage of the mix volume that the magnetite aggregate accounted for.

Specimens of eight cubes of 100 mm sides and one cylinder of 150 mm diameter and 300 mm height were prepared from each series. The cubic specimens were used in the compressive strength test, and the cylinders were cut into ten specimens as 25 mm thick slices. These slices were used to test the thermal properties and to determine the density of the material in the water-saturated state and in the dry state. All prepared specimens were matured in water until they were 28 days old. After this time, the cubic specimens were subjected to a compressive strength test, and the cylindrical specimens were cut into slices, which were again placed in water until thermal properties testing began.

### 2.2. Methods

Testing of the thermal properties of cementitious composites was conducted using a non-stationary method with the ISOMET 2114 (Applied Precision, Bratislava, Slovakia) portable handheld measuring instrument for direct measurement of heat transfer properties. This instrument conducts a two-stage measurement, during which two thermal parameters of the material under test are directly determined: thermal conductivity and thermal diffusivity. Based on the measured values of these parameters, the device calculates and additionally provides a value for the volumetric heat capacity. From this, the specific heat value can then be calculated if the density of the material is known. A more detailed description of the test procedure can be found, among others, in papers [9,10,26], where the same device and test methodology were used.

Thermal property tests were carried out on specimens in two states of water saturation: at full saturation and after drying the specimens to a constant weight at 65 °C. The drying temperature was deliberately chosen to limit the possibility of cracks forming in the specimens [27]. In addition, the temperature adopted corresponds to the average temperature to which the concrete of a nuclear reactor’s biological containment heats up [28,29]. The thermal properties were tested in two states of water saturation, as a material fully saturated with water has the highest thermal conductivity. In contrast, testing the specimens at the temperature to which the concrete in the biological shield structure heats up allows the thermal conductivity of the material to be estimated during prolonged normal operation of the reactor. There are two limit values. The first of these (at full water saturation) is the limit regardless of the circumstances of the material’s operation, and the second is the limit in this application.

The density of the hardened cementitious composites was determined by hydrostatic weighing of the specimens used to test their thermal properties. By weighing saturated specimens immersed in water directly on the scale, their volume was determined using Archimedes’ law. Subsequently, classical weighing of specimens dried to a constant weight allowed the density values in both saturation states to be calculated.

The porosity of the specimens was determined by the mass loss due to water evaporated during drying. The volumes of the specimens determined during the density measurement were used in the porosity calculations. From the mass loss, the volume occupied by the water evaporated from the material specimen was calculated. This volume was then divided by the volume of the specimen to obtain the porosity value. Due to the assumptions made regarding the drying process of the specimens (achieving a constant mass at a given temperature), the value obtained is not the total porosity, but the open porosity for water evaporable after long-term heating the material to 65 °C. However, as this parameter directly influences the obtained values of the thermal parameters of the tested composites, and to increase the readability of the article, in the following, the term porosity will be used simply to denote the determined parameter defined as described above.

The results obtained were illustrated in column graphs by comparing all the series studied in each graph. This made it possible to compare the results obtained with the different aggregate fractions with the CM40 and CM60 series, which can serve as reference series against them due to similarities in composition. The results obtained from saturated specimens and specimens dried to a constant weight were also summarised together. In addition to basic statistical analyses, which included the calculation of mean values, median values, and measures of the spread of the results, violin plots of the thermal parameter values obtained were also made. These made it possible to illustrate the distribution of the results, which allowed an in-depth analysis and additional conclusions to be drawn.

As an auxiliary test to assess the quality of the cementitious composites, a test of their compressive strength was conducted. The test was performed in accordance with PN-EN 12309-3 [30] on eight cubic specimens with an edge of 100 mm. The load increment in the test was 0.5 MPa/s. The tests were performed 28 days after the preparation of the mixtures. The specimens were stored in water until the test.

## 3. Results

### 3.1. Compressive Strength, Density, and Porosity

**Figure 1:**
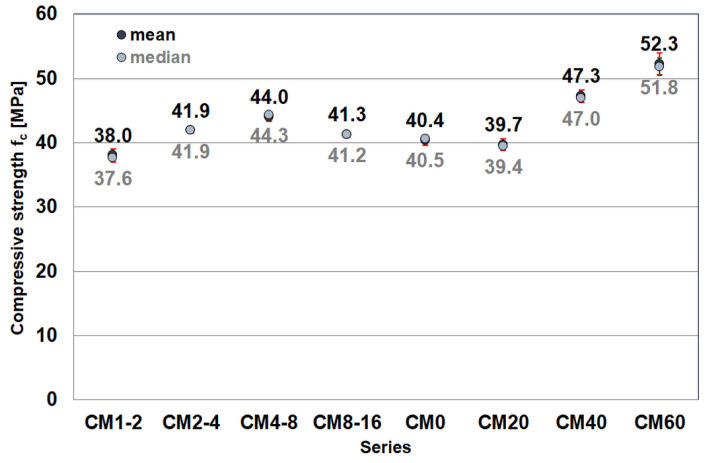
Mean values and median values of compressive strength of specimens of the tested cementitious composite.

Figure 1 shows the results of the compressive strength test. Due to the variation in the results obtained, it was decided to calculate both the mean value of the strength and the median, which is a robust parameter that is resistant to outliers found among the results.

In the series of concretes containing the same amount of magnetite aggregate but differing in particle size (CM1-2÷CM8-16), there is a clear increase in compressive strength with an increase in grain size up to 4–8 mm (44 MPa). Subsequently, in the concrete with a particle size of 8–16, there was a decrease in strength (41.3 MPa). On the other hand, in the series of concretes containing all fractions of magnetite aggregate but in varying amounts (CM20–CM60), an increase in compressive strength is observed with an increase in the content of magnetite aggregate. From 39.7 MPa for CM20 to 52.3 MPa for CM60.

**Figure 2:**
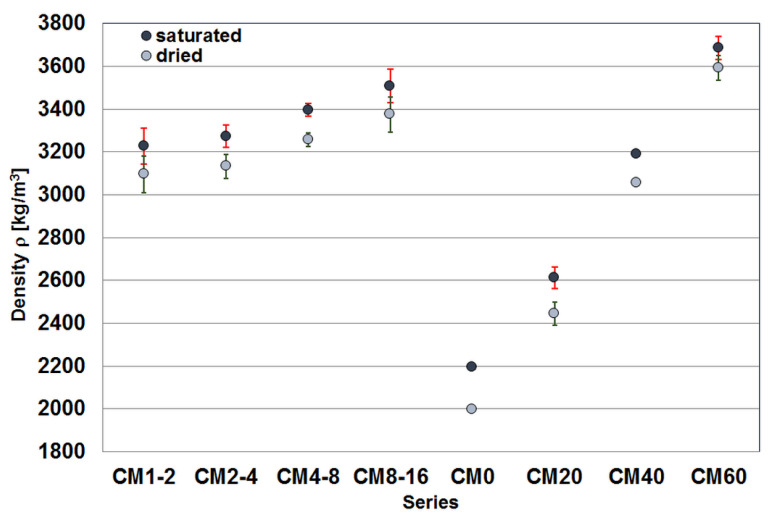
Median values of the density of the specimens of the tested cementitious composite at full water saturation and after drying to a constant mass at 65 °C.

**Figure 3:**
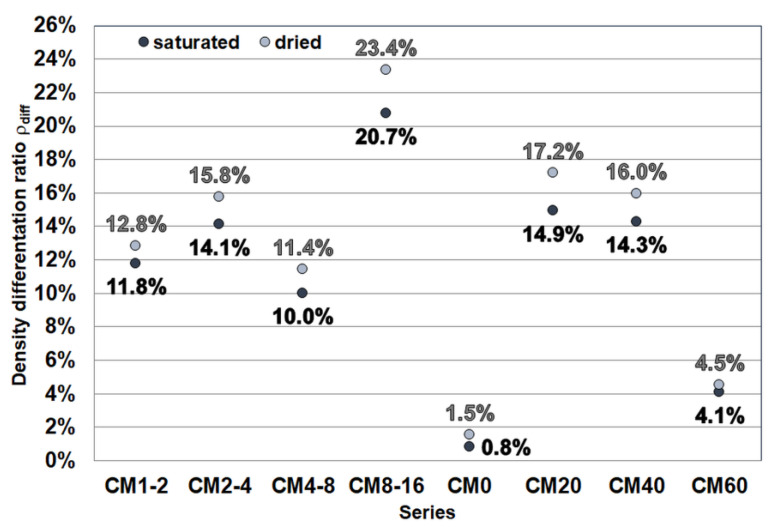
Values of the parameter determining the variation in the density of the composite in the specimens (description in text).

Figure 2 and Figure 3 show the median values of the test results for the density of the tested cementitious composites and a measure of the variation of this value. The error bars in Figure 2 represent the median average deviation. The density of the material with an equal volume share of magnetite aggregate (CM1-2 ÷ CM8-16), shows quite significant fluctuations, with a clear increase with the grain size of the aggregate. The lowest density was achieved by composite CM1-2 (3250 kg/m^3^) and the highest by composite CM8-16 (3550 kg/m^3^). However, significant differences in density are observed among composites containing varying amounts of magnetite aggregate. Composite CM20 exhibited the lowest density (2600 kg/m^3^), while composite CM60 had the highest density (3700 kg/m^3^).

Due to the significant difference in density between the cementitious matrix and the magnetite aggregate, the components of the mixture tend to segregate both during composite preparation and mould filling. As a result, the heavy magnetite aggregate moves downward, and its volume proportion in the upper part of the specimen slightly decreases. This leads to a variation in density over the height of the specimen, as illustrated in Figure 3.

A measure of the variation in density values was obtained by dividing the calculated density value of the specimen slice cut first from the top by the density of the slice cut first from the bottom. The result expressed was then subtracted from 1 and expressed as a percentage according to Formula (1):

ρ_diff_ = (1 − ρ_t_/ρ_b_)·100 [%], (1)

where: ρ_diff_—density differentiation ratio, ρ_t_—density of the top slice of the specimen, ρ_b_—density of the bottom slice of the specimen.

The density differentiation ratio ρ_diff_ calculated in this way has a higher value the greater the difference in density of the material layers being compared in the test specimen. to obtain an index that takes on the value of 0% when there is no variation in the density of the material.

The smallest variation was noted for the specimen of the CM0 series, which contains no magnetite aggregate. The very low variation in density is also shown by the CM60 series specimen. In this series, a superplasticiser was used due to the significant deterioration in workability. However, despite the change to a slightly more fluid consistency, there was no significant stratification of the mix components, which can be explained by the synergistic effect of the high proportion of magnetite aggregate in the mix volume and its still dense consistency (despite the addition of the superplasticiser).

**Figure 4:**
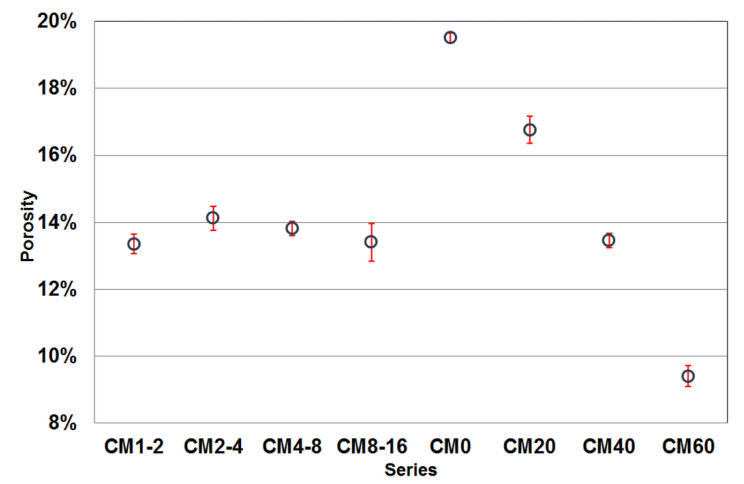
Median porosity values of the tested cement-based composite specimens.

Figure 4 shows the results of the porosity calculation of the specimens from the different series. The porosity of the series with an equal content of magnetite aggregate shows some variability. The series with the finest and coarsest fraction of magnetite aggregate (CM1-2 and CM8-16) have the lowest porosity, which is about 13.5% and is approximately the same as the porosity of the CM40 series. For the other two series, the porosity is slightly higher, at around 14%. However, these differences are not significant given the variability of the results.

### 3.2. Measured Thermal Parameters

#### 3.2.1. Thermal Conductivity

**Figure 5:**
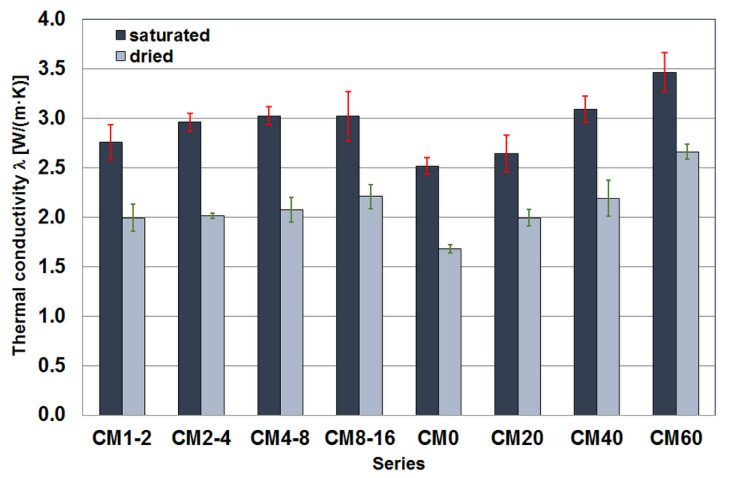
Median thermal conductivity values of the tested cement composite specimens.

The results of thermal conductivity tests on cement composites with magnetite aggregate are shown in Figure 5. The median values and median average deviation are given for specimens saturated with water and dried to constant mass at 65 °C. The results obtained for the series with equal content of magnetite aggregate do not differ significantly from each other, although a trend of increasing conductivity values with increasing grain size of the aggregate can be observed. This trend is apparent in both water-saturated and constant-mass dried specimens, although the pattern is slightly different in both cases.

The results obtained from tests of the series with different proportions of magnetite aggregate indicate a relationship between the amount of this aggregate and the thermal conductivity value obtained. As expected, increasing the proportion of magnetite aggregate, which has a higher thermal conductivity than the cement matrix, leads to an increase in the thermal conductivity of the composite. This is not a linear relationship over the entire range and is slightly different for water-saturated samples and samples dried to constant mass. However, in both cases, the difference between the lowest value obtained for the CM0 series and the highest value measured for the CM60 series is approximately 1 W/(m·K).

#### 3.2.2. Thermal Diffusivity

**Figure 6:**
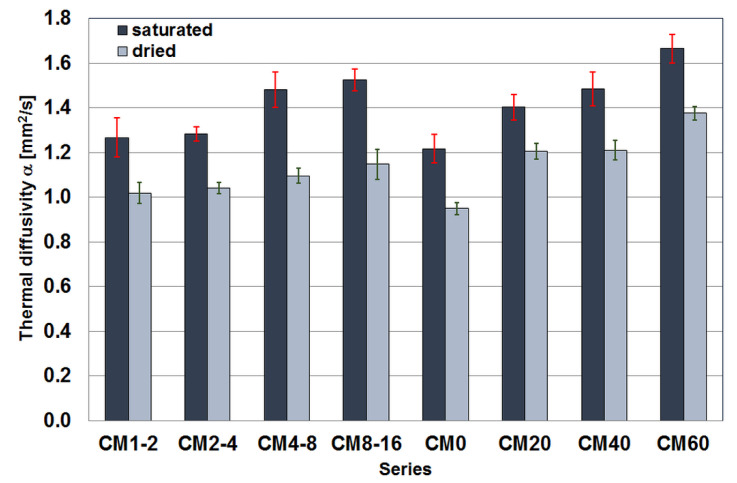
Median thermal diffusivity values of the tested cement composite specimens.

The results of the thermal diffusivity tests are shown in Figure 6. The results are the median of the values obtained from tests on individual samples that were tested in a fully water-saturated state and after drying to a constant mass. In the case of samples with a constant content of magnetite aggregate tested in a fully water-saturated state, a division into two groups of two series was outlined. In the composite samples with finer fractions, i.e., the CM1-2 and CM2-4 series, the median thermal diffusivity values obtained are practically equal at 1.27 mm^2^/s and 1.28 mm^2^/s, respectively. For the other two series, namely CM4-8 and CM8-16, the values are, respectively, 1.48 mm^2^/s and 1.53 mm^2^/s, i.e., not significantly different from each other. In contrast, the differences between the two groups of samples are significant.

When testing dry samples from the same composite series, the division between the two groups is much less pronounced due to the much smaller difference in thermal diffusivity values between the two groups of the series. While this reached 0.20 mm^2^/s for the wet samples, it dropped to 0.06 mm^2^/s for the dry samples and is statistically insignificant. The median thermal diffusivity values were 1.02 mm^2^/s, 1.04 mm^2^/s, 1.10 mm^2^/s, and 1.15 mm^2^/s for the dry specimens in the CM1-2, CM2-4, CM4-8 and CM8-16 series, respectively.

Investigations of the thermal diffusivity of the composite samples with variable magnetite aggregate content arranged themselves in an increasing trend for both the saturated samples and the samples dried to a constant mass. In both cases, this upward trend has stalled somewhat between the CM20 and CM40 series samples. With the saturated samples, this inhibition is less, although still pronounced. When fully saturated samples were tested, values of 1.22 mm^2^/s, 1.40 mm^2^/s, 1.48 mm^2^/s, 1.69 mm^2^/s were obtained for the CM0, CM20, CM40 and CM60 series, respectively. For the dried samples, the median thermal diffusivity values obtained for the CM20 and CM40 series are the same at 1.21 mm^2^/s, and for the CM0 and CM60 series, the values are 0.95 mm^2^/s, 1.38 mm^2^/s, respectively.

### 3.3. Calculated Thermal Parameters

#### 3.3.1. Volumetric Heat Capacity

**Figure 7:**
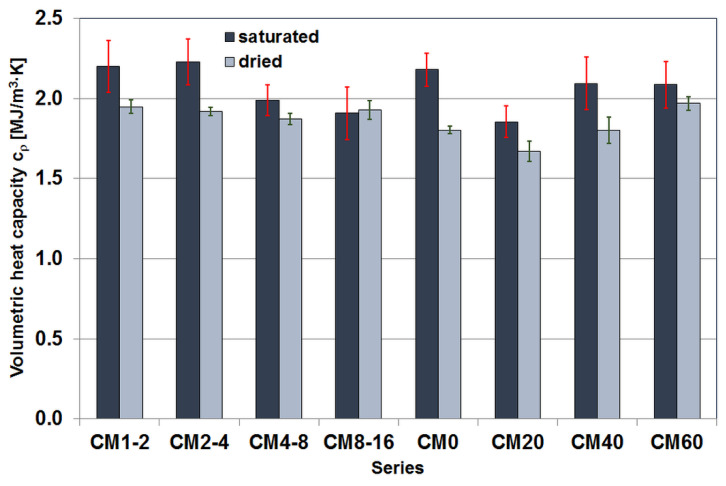
Median volumetric heat capacity values of the tested cement composite specimens.

The volumetric heat capacity values were continuously calculated by the measuring device based on the tested thermal conductivity and thermal diffusivity values as the result of dividing the former by the latter. Figure 7 shows the medians of the volumetric heat capacity values calculated in this way.

In cementitious composites with a fixed proportion of magnetite aggregate, the calculation results for saturated specimens largely replicate the division into two series groups seen earlier in the thermal diffusivity test results. In the case of dried samples, the median results obtained are far less differentiated, and the division into two series groups has disappeared. In addition, for the CM8-16 series, the median for the dried samples is larger than the median results of the saturated samples. However, this mutual configuration of results should be treated with great caution due to the high variability of the calculation results obtained for the saturated samples.

For composites with different proportions of magnetite aggregate, the calculation results do not form a clear trend. In the case of saturated samples, a decrease in the median value of the volumetric heat capacity can be seen first, followed by an increase and then stabilisation. The changes are similar for dried samples, with the difference that there is no stabilisation phase, but the median value after the initial decrease only shows an increase.

#### 3.3.2. Specific Heat

**Figure 8:**
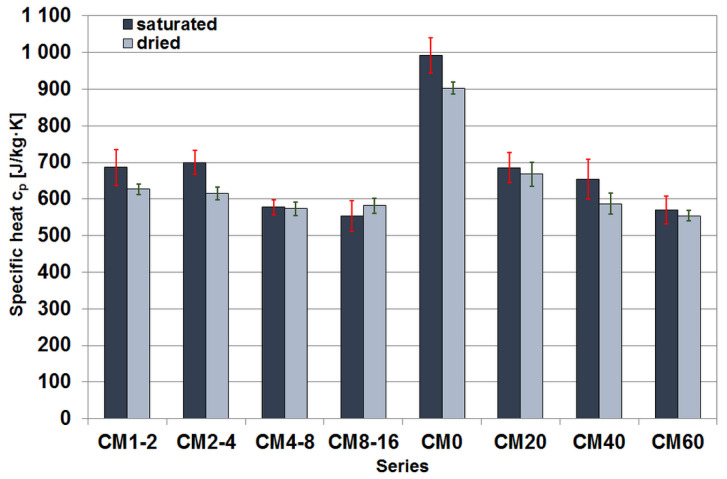
Median specific heat values of the tested cement composite specimens.

The specific heat was calculated from the results obtained from the volumetric heat capacity and density measurements of the composites. The values were calculated individually for each sample, and then the median and median average deviation were calculated in each case based on the results obtained, as shown in Figure 8.

In the case of series in which different fractions of magnetite aggregate were used in the same amount, the results formed a similar pattern to the volumetric heat capacity measurement values. This parameter decisively influenced the obtained specific heat values due to the slight differences in density between the series in this group of composites.

In the case of the series with different magnetite aggregate content, an exceptionally large decrease in the specific heat value is seen between the CM0 series and the other series, where the specific heat value still decreases slightly with increasing magnetite aggregate content. This decrease cannot be attributed solely to the difference in density, which is obvious when Figure 8 is juxtaposed with Figure 2. In the case of saturated specimens, this is the result of a slight increase in thermal conductivity when magnetite aggregate is added to the cement matrix and, at the same time, a much greater increase in thermal diffusivity. In contrast, in the case of dry samples, where the increase in conductivity values was more pronounced, the thermal diffusivity also increased to an even greater extent compared to the wet samples.

## 4. Discussion and Summary

### 4.1. Compressive Strength, Density and Porosity

The analysis of the compressive strength results reveals two discernible trends. Firstly, there is a general increase in the strength of the cement composite as the grain size of the magnetite aggregate increases, except for the CM8-16 series. This deviation aligns with the established pattern where the strength of cementitious composites typically decreases with larger aggregate grain sizes. This suggests that the observed increasing trend for composites with finer aggregate grain sizes may be influenced by other factors. However, Mehmetoğulları et al. [31] showed that the strength of cement-based composites with well-graded fine aggregate increased as the grain size increased. Gharieb et al. [22] showed that the presence of fine magnetite aggregate increased the 7-day, 28-day and 90-day compressive strengths by 23%, 12% and 9%, respectively, compared with magnetite concrete containing local fine sand.

Secondly, there is a notable increase in the strength of the cement-based composite with a higher content of magnetite aggregate, even with a significant reduction in the amount of cement. However, this effect is not evident when comparing the results of the CM0 and CM20 series, indicating that only when the proportion of magnetite aggregate exceeds 20% by volume does it significantly enhance the composite’s strength. The observed linear increase in strength with an increase in aggregate proportion can be estimated at about 0.31 MPa per percentage point. This observation aligns with findings reported by other authors [20,32]. Horszczaruk et al. [20] showed that the use of magnetite aggregate in concrete mixtures significantly improved mechanical properties when concrete was subjected to temperatures up to 450 °C. Ouda [32] tested high-performance heavyweight concretes, including concrete containing magnetite aggregate, and found that they exhibited maximum compressive strength.

In concrete (CM1-2÷CM8-16), the increase in density with the grain size of the aggregate is visible. This can be explained by the decreasing specific surface area of the magnetite aggregate, which means a decrease in the volume of ITZ between the aggregate and the matrix throughout the specimen volume. Due to the relatively poor wettability of magnetite in the presence of calcium ions [33], it is expected that the ITZ between the magnetite aggregate and the cement matrix has a significantly higher porosity than the cement matrix, as confirmed by Dąbrowski et al. [34]. They analysed the microstructure of heavy concrete and showed the discontinuity between the magnetite aggregate and the cement matrix. On the other hand, Zalegowski et al. [35] demonstrated that in concrete with magnetite aggregate, grains due to dust present on their surface negatively affected the properties of the ITZ.

The trend in density change of the composite with an increasing proportion of magnetite aggregate is upward, and the increase is roughly proportional to the increase in the volume proportion of the aggregate, as expected [32,34,35].

The greatest diversity in porosity was found in cement composite series with different magnetite aggregate contents. This is due to the significant difference between the porosity of the cement matrix, which can reach 20%, and the porosity of the magnetite aggregate, which, according to [36], is approximately 2.2%. The trend formed by the results of the series with increasing magnetite aggregate content is as expected and arranged in an almost proportional decreasing relationship. Comparing the two groups of series, it can be concluded that the series with the same content of magnetite aggregate have a higher porosity than would be expected. Given that they contain 50% of this aggregate, their theoretical porosity should be around 11.5%. However, it should be considered that the composition of the cement matrix in these series is slightly different. It contains more sand, which can affect its porosity. Gharieb et al. [22] revealed in their research that the fine magnetite aggregate absorbed more water than local sand. Besides, limiting the grain size of the magnetite aggregate to one fraction may also result in an increase in the porosity of the resulting composite, although the influence of this factor is, in the authors’ opinion, less than that of the proportion of the cement matrix components.

Research on the influence of magnetite aggregate on concrete porosity was conducted, among others, by [37,38]. Jóźwiak-Niedźwiedzka et al. [37] showed that the total pore volume evaluated by MIP was influenced by the content of magnetite aggregate in concrete. The relationship between the relative content of magnetite aggregate and the total pore volume was found. The total pore volume was linearly decreasing with the increasing content of magnetite aggregate. Lee and Kwon [38] showed reduced porosity with increasing both magnetite aggregate and steel powder.

### 4.2. Thermal Conductivity

**Figure 9:**
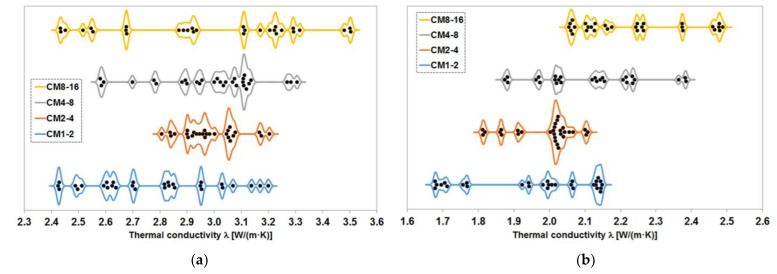
Distribution of the results of the measurement of the thermal conductivity coefficient of the series with equal content of magnetite aggregate: (**a**) samples fully saturated with water; (**b**) samples dried to constant mass at 65 °C.

Figure 9 shows the distribution of the thermal conductivity test results of the series with equal proportions of magnetite aggregate. Of the results obtained from the fully water-saturated samples, three measurements from the CM2-4 series, which obtained values below 2.1 W/(m·K), were excluded. The results of this series, as can be seen in Figure 9a, are clustered between approximately 2.8 W/(m·K) and approximately 3.2 W/(m·K), so the excluded results can undoubtedly be classified as outliers. A similar exclusion was made in the results obtained on samples dried to constant mass. In this case, three values of approximately 1.1 W/(m·K) were obtained in the CM4-8 series, while the other results range from 1.8 W/(m·K) to 2.4 W/(m·K), so these three results should also be considered outliers.

When analysing the results obtained on fully saturated samples, as the grain size of the aggregate used increases, the range within which the thermal conductivity coefficient values fall also increases. At the same time, the maximum value obtained from the measurements increases and the minimum value decreases. In this way, a change occurs in the results obtained for the series with magnetite aggregate fractions above 2 mm. The CM1-2 series does not fit into this pattern, although the maximum measured value of the thermal conductivity coefficient is slightly lower than in the CM2-4 series.

A possible explanation for this is the increased inhomogeneity of the composite arising primarily during the compaction of the mixture in the moulds on the vibrating table. The larger aggregate grains in the mixture have a smaller total surface area and, therefore, lower wettability, resulting in a more fluid mixture. Larger grains are also more susceptible to sedimentation, both under the influence of gravity and due to inertial forces during the compaction of the mix. Increased segregation increases the likelihood of areas with varying proportions of magnetite aggregate, i.e., both areas where there is more magnetite than average in the whole specimen and areas where there is less. In the former areas, the thermal conductivity is significantly higher, and in the latter, it is lower. This effect becomes more pronounced with coarser grains of the magnetite aggregate fraction used.

The relationship is completely different for samples dried to constant mass at 65 °C. Here, there is a noticeable trend towards an increase in both the minimum and maximum values of the thermal conductivity coefficient with increasing grain size of the aggregate. At the same time, the increase in both values varies, with even a reversal in the cases of the CM1-2 and CM2-4 series. It is worth noting, however, that the results of the CM2-4 series are very concentrated around the median and fall within a significantly shorter range than those of the other series.

When analysing the changes in the distribution of the results obtained, it is worth bearing in mind that the samples were tested in a certain state of equilibrium in terms of moisture content. Drying allowed them to be brought to a constant weight, but this does not mean that all the contained water was removed. It remained in some quantity, especially in the pores of the cement matrix, including capillary and closed pores. Because of the fact that, in the case of specimens with a larger grain size of the magnetite aggregate, there is a greater material variation in terms of the proportion of matrix and aggregate, the different trend in the minimum values obtained in the tests of the individual series can be explained. Clusters of larger cement matrix volumes with more partially water-filled pores provide better thermal conductivity. Thus, despite the greater variation in composite structure, low thermal conductivity values are less likely to be obtained. Larger matrix clusters between the larger aggregate grains provide slightly more water retention and thus have the effect of increasing thermal conductivity relative to areas more drained of water during the drying process.

**Figure 10:**
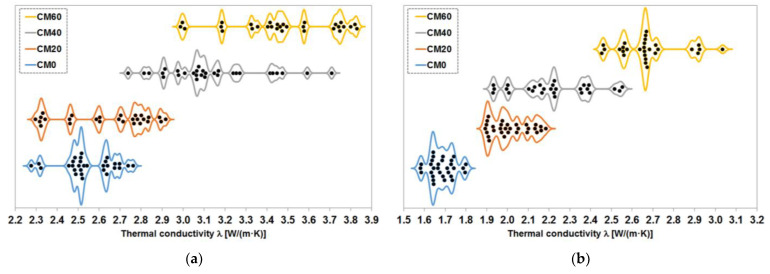
Distribution of the results of the measurement of the thermal conductivity coefficient of the series with different contents of magnetite aggregate: (**a**) samples fully saturated with water; (**b**) samples dried to constant mass at 65 °C.

The distributions of the results of the measurements of the thermal conductivity coefficient on the samples of the series with different contents of magnetite aggregate are shown in Figure 10. As outliers, the three measurements of the samples fully saturated with water in the CM20 series and the three measurements of the samples dried to a constant mass in the CM40 series are not shown there. The measurements obtained in their case were clearly underestimated.

The distribution of results for the fully water-saturated samples shows that an increase in the proportion of magnetite aggregate from 20% by volume to 40% resulted in a very large increase in the maximum values of the thermal conductivity coefficient obtained, after which a further increase in this proportion to 60% resulted in a slight increase. The minimum values are similar, with a negligible difference between the CM0 and CM20 series and a significant increase for the CM60 series compared to the CM40 series. This indicates the possibility of the existence of a certain threshold share of aggregate, beyond which the thermal conductivity of a composite material composed of phases with quite different thermal resistances increases sharply. On the other hand, it can be noted that although the ranges of the thermal conductivity results for the CM0 and CM20 series are similar, in the case of the latter series, we have a rather clear shift in the point around which most of the results are concentrated. Whereas in the CM0 series it was around 2.5 W/(m·K), in the case of the CM20 series this point is in the vicinity of the value of 2.8 W/(m·K). Thus, intuitively, the addition of magnetite aggregates already at 20% by volume has a noticeable effect on the distribution of results, although due to the significant shift of some of the results towards the minimum value, the effect is less well expressed.

The situation looks different for samples dried to constant mass, where there is an almost linear upward trend in both the maximum and minimum values of the thermal conductivity coefficient. This is only disturbed in the case of the minimum value of the CM40 series, which is practically at the same level as for the CM20 series. However, when analysing the values around which the most results accumulate in each series, it is easy to see that, in the case of the CM40 series, it is shifted in relation to the CM20 series by a rather significant value, which confirms the trend of an increase in the thermal conductivity of the composite with an increase in the proportion of magnetite aggregate. Such a patterned trend is in line with both intuition and expectations, so a more detailed analysis of it seems unnecessary.

### 4.3. Thermal Diffusivity

**Figure 11:**
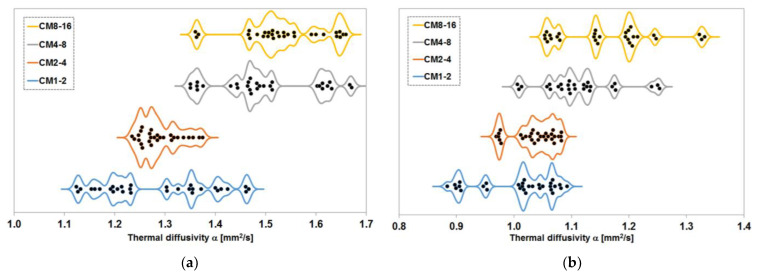
Distribution of the results of the measurement of the thermal diffusivity of the series with equal content of magnetite aggregate: (**a**) samples fully saturated with water; (**b**) samples dried to constant mass at 65 °C.

The distribution of the thermal diffusivity results obtained in the measurements of the samples of the series with equal content of magnetite aggregate is shown in Figure 11. Three values obtained on the samples of the CM2-4 series in the fully water-saturated condition, three values for the CM4-8 series when tested on samples dried to a constant mass and one result of the CM8-16 series obtained on a sample in the same condition were identified as outliers. In the case of the CM2-4 series, the rejected results were significantly higher than the others, and in the remaining cases, they were lower.

Analysing the obtained distributions of results, the minimum measured value of thermal diffusivity increases with increasing grain size of the magnetite aggregate. In the case of samples fully saturated with water, this increase slows down in the last series, i.e., CM8-16. In tests on dried specimens, no such effect occurred. As far as the maximum value obtained is concerned, it shows remarkably similar variability between series. With one exception. Due to the more pronounced lower range of variability of the measurement results in the case of series CM2-4, this series does not fit into the trends described above for the change in the maximum measured value.

The analysis of the values around which the highest number of results are clustered leads to similar conclusions as the analysis of the medians. In the case of fully water-saturated samples, a division into two groups of series is apparent: those with finer aggregate fractions and those with coarser fractions. In the former group, the results are concentrated around lower values than in the latter. In the case of samples dried to a constant mass, this division is less apparent, although the first two series, i.e., CM1-2 and CM2-4, have most of the results concentrated in an almost identical range of values. This correlates well with the density results shown earlier in Figure 2 and indicates an indirect relationship between aggregate grain size and thermal diffusivity results, in which the density of the composite plays a key role.

**Figure 12:**
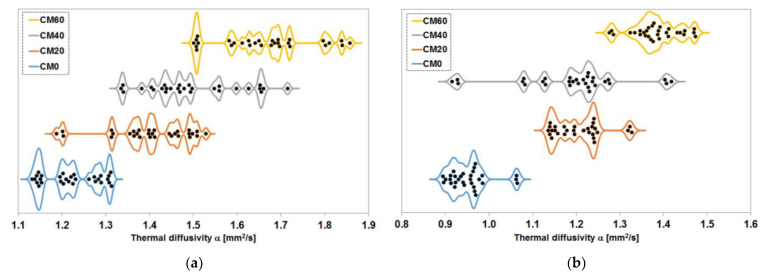
Distribution of the results of the measurement of the thermal diffusivity of the series with different contents of magnetite aggregate: (**a**) samples fully saturated with water; (**b**) samples dried to constant mass at 65 °C.

The distribution of the results of the thermal diffusivity test for the series with different proportions of magnetite aggregate is shown in Figure 12. Analysing the results obtained for the samples fully saturated with water, the minimum and minimum values in the CM20÷CM60 series form a trend in which, as the volume proportion of aggregate increases, both of these distinctive values increase proportionally to it. In the case of the CM0 series, on the other hand, both the minimum value and the maximum value of thermal diffusivity obtained from the measurements are admittedly lower than in the case of the CM20 series, but they do not follow the trends identified above. The maximum value is significantly lower in this series than in the CM20 series, while the decrease in the minimum value is less than that between successive series with increasing proportions of magnetite aggregate. Nor do the values around which most values are concentrated form such a proportional trend. What is more, it is difficult to even pinpoint such values, and one should rather refer to ranges in this case. Thus, in the CM20 and CM40 series, the ranges practically coincide, only to shift markedly towards lower values in the CM0 series and towards higher values in the CM60 series.

The results obtained on specimens dried to constant mass show similarity to those obtained on specimens fully saturated with water only regarding the compliance of the concentration intervals of most results. In this case, similarly, the interval first shifts significantly towards higher values if we compare the CM0 and CM20 series with each other. Then, in the case of the CM40 series, it can be considered as not changing significantly, only to shift again clearly towards higher values of thermal diffusivity when moving on to the CM60 series. It is also noticeable that the results are generally more concentrated, as in the case of the CM40 series, if the six results at the two extreme positions on the graph were considered outliers.

### 4.4. Volumetric Heat Capacity and Specific Heat

The results of the volumetric heat capacity and specific heat were not analysed in terms of their distribution. This is mainly due to the indirect way in which they were obtained (calculations from measured parameters), which favours the accumulation of possible errors or inaccuracies. In addition, the volumetric heat capacity values do not follow a clear trend, and an analysis of their distribution would not add anything new.

Based on an analysis of the median values, it can be concluded that, in the case of series with a constant volume proportion of magnetite aggregate, the volumetric heat capacity of samples dried to a constant mass is at a very similar level, and it can be assumed that the variations between individual series are negligible. In the case of samples fully saturated with water, the variation in this parameter is correlated with the variation in density of the composite and indicates the significant contribution of the water present in the pores of the material to the volumetric heat capacity value, which is due to its high volumetric heat capacity of 4.2 MJ/(m^3^·K).

For specimens with different proportions of magnetite aggregate, an initial decrease in volumetric heat capacity between the CM0 and CM20 series is noticeable, followed by an increase as the proportion of aggregate increases. This trend is less pronounced for the wet samples. This initial decrease can be explained by the much greater increase in thermal diffusivity when magnetite aggregate is added to the cement matrix in relation to the increase in thermal conductivity. As the proportion of magnetite aggregate increases, the volumetric heat capacity also starts to increase, which is due to the rather high value of this parameter for magnetite (about 3.4 MJ/(m^3^·K) according to [36]).

The variability of the median values of specific heat for the series with variable magnetite aggregate content does not show qualitative deviations from the expected trend into which these results should fall. As the proportion of magnetite aggregate increases, the specific heat value decreases. From a qualitative analysis perspective, the high value of this parameter for the CM0 series in relation to the value calculated for the CM20 series is surprising. Given that the specific heat values were obtained in the least direct way, these results should be treated with caution and reserve. It is most likely that the results of the CM20 series are underestimated and that the trend is set by the values obtained from the calculations in the other series (while taking here into account the rather high variability of the results).

Similar reservations must be considered for the results of the series with a fixed proportion of magnetite aggregate. In the case of the dried specimens, however, it can be assumed with some approximation that the results obtained are on a similar level, as their actual variability is higher than that obtained from the calculations. In contrast, in the case of fully water-saturated samples and series with finer fractions of magnetite aggregate, the higher specific heat values reflect the influence of the water contained in the samples with increased porosity. This explanation is indicated by the lower composite density values in the two series.

## 5. Conclusions

The results obtained in this study indicate that the thermal properties of cement-based composites with magnetite aggregate generally depend on the content of this aggregate in the material. Such a statement would be a truism, were it not for the fact that this relationship did not turn out to be simple and direct. The additive parameters, such as specific heat and volumetric heat capacity, show clear deviations from a simple correlation with the magnetite content of the material and do not reach the values they would assume if they were to follow the mixture rule.

Moreover, the results obtained indicate that it is not only the amount of magnetite aggregate contained in the composite that influences its thermal performance but also its grain size. Such a conclusion can be drawn by analysing the results of series with the same volume proportion of magnetite, but with different grain sizes.

The quantitative results can be summarised in consecutive points:

1. The measured value of thermal conductivity of material with the same volume of magnetite and different grain size is in the range 2.76–3.03 W/(m·K) and 2.00–2.21 W/(m·K) at full water saturation and after drying to a constant mass, respectively. The differences are 10% and 11%, respectively, in relation to the lower value.
2. The measured value of thermal diffusivity of material with the same volume of magnetite and different grain size is in the range 1.27–1.53 mm^2^/s and 1.02–1.15 mm^2^/s at full water saturation and after drying to a constant mass, respectively. The differences are 20% and 13%, respectively, in relation to the lower value.
3. The calculated value of volumetric heat capacity of material with the same volume of magnetite and different grain size is in the range of 1.91–2.23 MJ/(m^3^·K) and 1.87–1.95 MJ/(m^3^·K) at full water saturation and after drying to a constant mass, respectively. The differences are 17% and 4%, respectively, in relation to the lower value.
4. The calculated value of specific heat of material with the same volume of magnetite and different grain size is in the range 553–700 J/(kg·K) and 573–627 J/(kg·K) at full water saturation and after drying to a constant mass, respectively. The differences are 26% and 9%, respectively, in relation to the lower value.
5. The measured values of thermal conductivity of material with 0%, 20%, 40% and 60% volume content of magnetite are 2.52 W/(m·K), 2.65 W/(m·K), 3.09 W/(m·K) and 3.47 W/(m·K) at full water saturation and 1.67 W/(m·K), 1.99 W/(m·K), 2.19 W/(m·K) and 2.66 W/(m·K) after drying to a constant mass, respectively.
6. The measured values of thermal diffusivity of material with 0%, 20%, 40% and 60% volume content of magnetite are 1.22 mm^2^/s, 1.40, mm^2^/s 1.48 mm^2^/s and 1.67 mm^2^/s at full water saturation and 0.95 mm^2^/s, 1.21 mm^2^/s, 1.21 mm^2^/s and 1.378 mm^2^/s after drying to a constant mass, respectively.
7. The calculated values of volumetric heat capacity of material with 0%, 20%, 40% and 60% volume content of magnetite are 2.18 MJ/(m^3^·K), 1.85 MJ/(m^3^·K), 2.09 MJ/(m^3^·K) and 2.09 mm^2^/s at full water saturation and 1.80 MJ/(m^3^·K), 1.67 MJ/(m^3^·K), 1.80 MJ/(m^3^·K) and 1.97 MJ/(m^3^·K) after drying to a constant mass, respectively.
8. The calculated values of specific heat of material with 0%, 20%, 40% and 60% volume content of magnetite are 992 J/(kg·K), 686 J/(kg·K), 654 J/(kg·K) and 570 J/(kg·K) at full water saturation and 903 J/(kg·K), 668 J/(kg·K), 587 J/(kg·K), 554 J/(kg·K), after drying to a constant mass, respectively.

The results obtained are worth verifying in further studies. The study of composites containing the same amount of coarse aggregate but two different types with varying proportions of magnetite and aggregate with thermal parameters close to those of the cement matrix seems to be an interesting direction. Also, numerical modelling of the heat flow in a material consisting of phases with significantly different thermal parameters and with different degrees of fineness of the dispersed phase may yield interesting conclusions.

## Figures and Tables

**Table 1:** Mix proportions of the analysed cement-based composites (in kg/m^3^).

Content/Parameter	CM 1-2	CM 2-4	CM 4-8	CM 8-16	CM0	CM20	CM40	CM60
Cement CEM II/B-V 32.5	360	360	360	360	600	480	360	240
River sand 0–2 mm	530	530	530	530	1325	1060	795	530
Magnetite 1–2 mm	2100	--	--	--	--	67	134	202
Magnetite 2–4 mm	--	2100	--	--	--	97	193	290
Magnetite 4–8 mm	--	--	2100	--	--	367	734	1101
Magnetite 8–16 mm	--	--	--	2100	--	309	618	927
Water	180	180	180	180	300	240	180	120
Superplasticizer	--	--	--	--	--	--	--	0.96
w/c	0.5							
Magnetite (total)	2100	2100	2100	2100	0	840	1680	2520
% mass of the magnetite	66.2%	66.2%	66.2%	66.2%	0.0%	32.1%	55.7%	73.9%
% volume of the magnetite	50%	50%	50%	50%	0%	20%	40%	60%

## Data Availability

The original contributions presented in the study are included in the article, further inquiries can be directed to the corresponding authors.
